# Classroom-comfort-data: A method to collect comprehensive information on thermal comfort in school classrooms

**DOI:** 10.1016/j.mex.2019.11.004

**Published:** 2019-11-07

**Authors:** Carolina M. Rodriguez, María Camila Coronado, Juan Manuel Medina

**Affiliations:** a University Piloto de Colombia, Bogotá, Colombia; b University of Los Andes, Bogotá, Colombia

**Keywords:** Classroom-comfort-data: a method to collect comprehensive information on thermal comfort in school classrooms, Data-collection methods in architecture, Children thermal comfort, Post-occupancy studies in educational buildings, Well-being in classrooms

## Abstract

Data from post-occupancy studies in real constructions have been instrumental in the development of mainstream thermal comfort standards for the built environment. However, there is growing evidence of the need to advance these standards, through more robust and comprehensive fieldwork records from a broader spectrum of geographies, climates, architectural characteristics and occupancies. It has been shown that the standards have limited suitability in environments such as educational buildings, as they were developed based mainly on adult subjects working in offices. The lack of guidance in data collection methodologies is also thought to require particular attention, as the accuracy of the assessment models relies significantly on the quality of the information gathered. This manuscript proposes a method to systematically acquire an extensive range of data specifically from school classrooms. The method seeks to improve current techniques as follows:

• The post-occupancy surveys suggested in mainstream standards focus mainly on the collection of physical and environmental parameters related to adult subjects. Classroom-comfort-data can be used to collect information not only on physical and environmental parameters but also on physiological and psychological aspects. It also includes tools tailored for occupants from different ages (7 years old and above).
• The assessment models suggested in mainstream standards employ between 2–5 parameters to predict thermal comfort ranges. The Classroom-comfort-data method is designed to gather up to 49 different thermal comfort parameters, which allow a more comprehensive evaluation of perception and preference, as well as adaptive strategies, social context, and cognitive and emotional appraisals.
• The existing surveys in the standards were formulated primarily for office environments in subtropical and temperate climates. The Classroom-comfort-data method can be adapted to fieldwork within different conditions of climate, building design, occupancy levels, and cultural contexts.

## Graphical abstract

**Figure:**
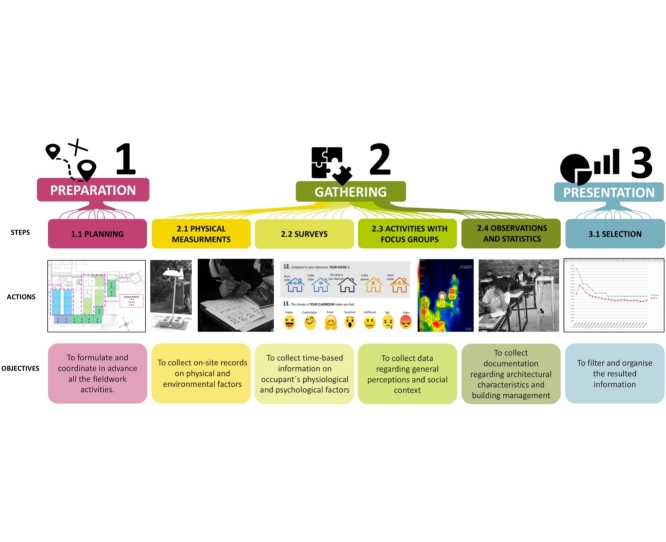

Specification Table

**Table:** 

Subject Area:	*Environmental Science*
More specific subject area:	*Thermal comfort in the built environment*
Method name:	*Classroom-comfort-data: A method to collect comprehensive information on thermal comfort in school classrooms*
Name and reference of original method:	*This method complements data collection techniques suggested in mainstream standards, such as the ANSI/ASHRAE Standard 55, the CSN EN* 15251 *and the ISO 7730.*
Resource availability:	*See Supplementary material*

## Methods details

Thermal comfort (TC) in the built environment is one of the most defining parameters influencing energy use, environmental quality and occupant satisfaction [1,2]. Research in this area is continuously growing, as the use of mechanical air conditioning can significantly impact on the increase of energy consumption in buildings and the production of waste. Thermally inadequate spaces can also lead to poor general health and wellbeing and contribute to the development of different physical and mental illnesses [1,3]. Yet evaluating TC is a complex task that involves the study of multiple interrelated physiological, psychological, and social factors [1,[4], [5], [6], [7], [8]]. The most widely used models to do this are the *static model* or *PMV* and the *adaptive model.* The former model, introduced by Fanger, P.O. in the 1970s, focuses on the study of set physiological parameters related to the heat exchange between humans and the environment. The latter model was based on the work of various authors including de Dear, R. Arens, E Nicol, F and Humphreys, M. during the 1990s. It includes the study of other dynamic parameters related to human behaviour and outdoor climate and it is suggested for the study of naturally ventilated buildings. Both models were developed with the support of the American Society of Heating and Air-Conditioning Engineers (ASHRAE) and became the foundation for standards such as the ANSI/ASHRAE Standard 55 [9], the CSN EN 15251 [10] and the ISO 7730 [11].

Although these models and standards have been adjusted over time, there is still no academic consensus on their feasibility or applicability within different contexts. Past research by the authors has found that the current mainstream standards are inadequate for the typical cultural, economic and environmental conditions in developing countries with tropical climates, as the information used for their development is based on other regions [12,13]. It is argued that the thermal neutrality resulting from the algorithms suggested by the standards is not necessarily the ideal setting for many occupants [[14], [15], [16]]. This is because the perception of neutrality can vary significantly between different climates and seasons [17,18] and between occupants according to age [15] gender [16] and cultural background [18]. Numerous studies report significant overlaps between the comfort levels predicted by the models and the actual data found during fieldwork [4,6,15,[19], [20], [21], [22], [23]]. Other literature highlights the complexity involved when assessing thermal comfort and the need for further development to understand all its interrelated aspects [1,[4], [5], [6], [7], [8],^24^]. In many reported cases, other types of occupant´s adaptations cannot be explained solely by the adaptations suggested in the current models [21,25]. Therefore, some authors advocate for a focal shift in thermal comfort research towards behavioural expressions of comfort, as lifestyles and control actions are thought to play a crucial part in energy use [5]. A deeper understanding of this subject is thought to require not only the study of physical or environmental parameters but also the evaluation of physiological and psychological information about comfort in the human body [26,27]. Related studies highlight the opportunities of personalised conditioning systems which are claimed to improve comfort while reducing the energy consumption, even though they are still not widespread in the building practice [7,28,29].

Additionally, a lack of connection between thermal assessment procedures and the architectural and constructional characteristics of buildings has been widely documented [30]. There is growing evidence to suggest the need to further confirm these models by more robust and comprehensive statistical and scientific data, gathered through post-occupancy studies [25,31,32]. Deficiencies in data collection and fieldwork methodologies are thought to require particular attention since the accuracy of the theoretical models relies significantly on the quality of the recorded data from real buildings [4,22,33].

The assessment of TC in schools is a particular concern, as it has been found that the conventional algorithms and benchmarks proposed by the current standards are not sufficiently accurate or adequate for educational buildings [15,34]. This is because the models were developed based on studies focusing primarily on office buildings occupied by adults. Research suggests that children tend to have different comfort levels as their metabolic rate, type of clothing, and level of activity varies with age [35]. It has been argued that children tend to be more sensitive to higher temperatures than adults with comfort being approximately 4 °C and 2 °C lower than the static and adaptive model predictions respectively [35]. Additionally, children are more prone to illness related to their environment, because their organs are smaller, more vulnerable and in the process of development [15,[36], [37], [38]].

There is still a general deficit of studies regarding children’s TC perception and little awareness of their importance [39,40]. Traditional fieldwork methods to record information are insufficient or unsuitable for children as the questions, language, and protocols are inappropriate for their age and development levels. In this context, the present work aims to contribute with a method, named *Classroom-comfort-data,* designed to gather comprehensive TC data in school classrooms. This method seeks to improve and complement the data-collecting techniques proposed in the existing standards by expanding on the following aspects:

• **Scope**: existing methods focus on collecting data on thermal sensation, preference and the feeling of comfort. However, they commonly ignored local traditions and cultural practices on climate adaptation. The proposed method allows gathering information on six key areas: 1. Evaluation; 2. Perception; 3. Preference; 4. Adaptive strategies; 5. Physical-cognitive context; and 6. Social context. This information supports the assessment of TC not just through the lenses of the *static* or *adaptive* models, but also via alternative theories on environmental satisfaction [41,42] or cognitive appraisal that centre on social and psychological relationships between sensation and evaluation [43].
• **Range**: The method has been designed to collect structured information from the building and from the occupants, as well as from the perspective of the data collector. This view is often underestimated or obtained informally, yet it can be of great contribution to TC assessment.
• **Adaptability**: The proposed strategies can be adapted to fieldwork within different conditions of climate, building design, occupancy levels, and cultural contexts.
• **Means**: Diverse tools are offered for every stage of the process.

## Method description

**Fig. 1:**
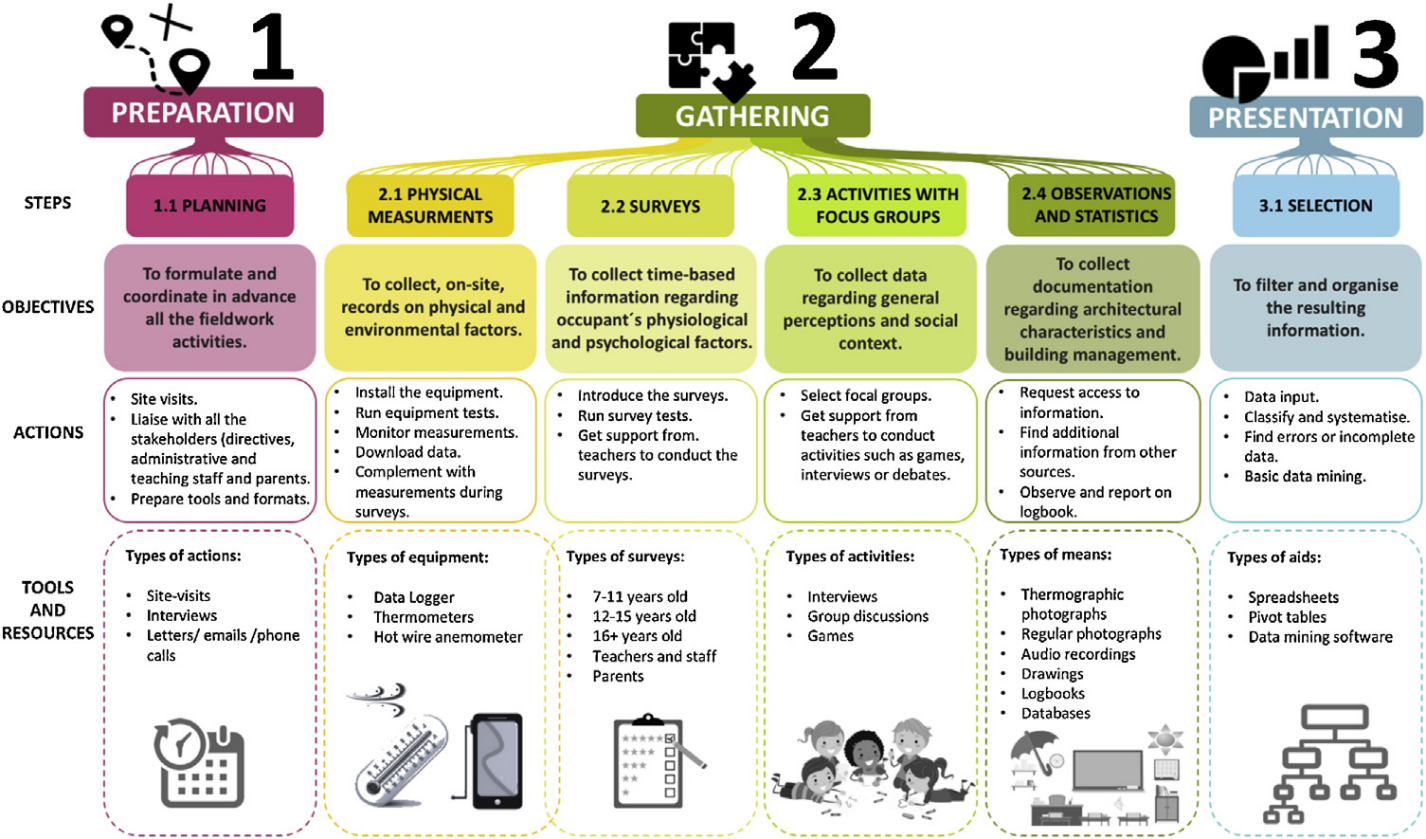
Structure of Classroom-comfort-data.

The method Classroom-comfort-data suggests the structure and stages for gathering a wide range of information during fieldwork to study the real thermal comfort conditions in school classrooms. It was tested and refined through two different case studies in schools in Bogota, Colombia. The final structure of Classroom-comfort-data comprises three consecutive stages: preparation, gathering, and presentation. Fig. 1 illustrates the various steps, objectives, actions, tools, and formats involved in each stage, which are described in more detail throughout this document (see Fig. 1).

### Preparation

Fieldwork and post-occupancy studies are generally costly, time-consuming and require considerable administrative efforts in advance. Therefore, it is vital to gather all the necessary information while on-site. The planning stage is often laborious due to the type and number of parameters which could be measured to study TC. These parameters can vary according to the model chosen for the assessment process. For example, some of the data needed to apply the static model is different from the data for the adaptive model or other alternative evaluation strategies. Depending on the case, information on certain parameters may not be needed or feasible to obtain. Therefore, some standards admit the simplification of parameters such as mean radiant temperature and airspeed, as well as the use of tables to calculate the metabolic rate and clothing insulation. However, it is argued that the more parameters are considered, the more comprehensive the understanding of TC could be.

Unfortunately, the general lack of clear guidelines on means and ways to obtain different types of data makes fieldwork planning more difficult. With this in mind, Table 1 was constructed for the present work based on an extensive literature review on parameters that potentially affect TC (see Table 1). This table lists and classifies 49 parameters into three main groups: 1. Physical and environmental factors; 2. Physiological factors and 3. Psychological factors. It also indicates which parameters need to be considered according to the assessment model, as well as the tools and formats suggested with the Classroom-comfort-data method. Extending on the importance or effect of each variable is out of the scope of this manuscript. However, references have been included to allow the reader exploring these issues further.

**Table 1:** Thermal comfort parameters that can be studied during fieldwork.

Thermal Comfort Parametres					Assessment MODELS		Ref Other Info	Proposed Method - Measuring Tool
		Variable		Unit/Type	Static	Adaptive		
Physical and Environmental Factors	Physical Space	Dry-bulb air temperature		°C	x	x		Dry bulb thermometer 10–40 °C range. ± 0.2 °C accuracy
		Globe temperature (GT)			x	x		Black globe thermometer Ø 150 mm. 10–40 °C range. ± 1 °C accuracy
		Mean radiant temperature (MRT)		°C	x	x		Derived from dry-bulb air temperature, globe temperature and airspeed.
		Operative temperature		°C	x	x		Derived from dry-bulb air temperature, mean radiant temperature and airspeed.
		Plane radiant temperature		°C			[9]	Surface temperature sensor. 10–50 °C range. ± 0.5 °C accuracy
		Prevailing mean outdoor temperature		°C		x		Portable weather station
		Airspeed		m/s	x	x		Hot wire anemometer 0.05–2 m/s range. ± 0.05 m/s accuracy
		Relative Humidity		%	x			Capacitive/resistive electronic hygrometer. 25–95 % range. ± 5% accuracy
		CO2 concentration		Parts-per-million			[44]	CO_2_ ppm meter
	Fieldwork Methodology	No. rooms/records		No. rooms			[4,22,46]	Observation logbook
		Equipment location		Coordinates and height				Observation logbook
		Date/time/season		DD/MM/YYY, winter, summer, spring, and autumn				Observation logbook
		Study type		Longitudinal or cross-sectional				Observation logbook
	Architectural Design	Envelope materials and building components		Material assemblies			[48,49]	Observation logbook
		Estimated insulating properties of building components		U Values				Dynamic simulations
		Space/room volume		m^3^				Dynamic simulations
		Space/room orientation /location		North, South, East, West, Ground floor, 1 st floor, etc.				Dynamic simulations
		Building use		Educational, residential, office, etc.				Survey - Observation logbook
		Building control		NV, MM, AC				Survey - Observation logbook
		Occupancy density		people/m^2^				Survey - Observation logbook
		Occupancy schedule		% time				Survey - Observation logbook
		Equipment and artificial lighting heat gains		W/m^2^				Dynamic simulations
		Equipment and artificial lighting usage		%				Survey - Observation logbook
		Energy consumption		Watts Per Day				General data collection
		Thermal-bridges		location				Thermal camera
	Local Context	Altitude		m a.s.l			[50]	General data collection
		Climatic/urban characteristics		K-G classification				General data collection
Physiological Factors	Occupant’s Physical Characteristics	Metabolic rate		met	x			Survey - Observation logbook
		Clothing insulation		clo	x			Survey - Observation logbook
		Gender		(M, F)			[15,51,53]	Survey - Observation logbook
		Age		(0–5, 6–				Survey - Observation logbook
		General health		Healthy, ill, other				Survey - Observation logbook
		Body heat		°C				Thermographic camera
		Weight		Kg				Scale
Psychological Factors	Occupant´s Behaviour	ADAPTIVE BEHAVIOUR	Environmental modifications to space	Open/close windows, change materials, add furniture, etc.			[41]	Survey - Observation logbook
			Behavioural adaptations	Dress warmly, remove clothes, hot/ cold drinks, etc.				Survey - Observation logbook
			Expectation´s adjustments	Get used to it, change expectation, etc.				Survey - Observation logbook
			Withdrawal from space	Temporarily leave the space, move out, etc.				Survey - Observation logbook
		SATISFACTION	Occupants’ thermal sensation Actual Mean Vote (AMV)	(1_7)		x		Focus group - interviews
			Predicted Mean Vote (PMV)	Temp/Humidity	x			Psychrometric charts
			Predicted Percentage of Dissatisfied (PPD)	%	x			Static model Charts
			Thermal sensation vote (TSV)	(+3 _ −3) seven-point scale		x		Survey - Observation logbook
			Thermal comfort/satisfaction vote (TCV)	(0_−3) seven-point scale		x		Survey - Observation logbook
		APPRAISAL	Conduciveness /goal	Promotion, interference, no impact			[28,43,54]	Survey - Focus group - Observation logbook
			Causality	Avoidable, unavoidable				Survey - Focus group - Observation logbook
			Perceived control/responsibility	Responsibility, agency.				Survey - Focus group - Observation logbook
			Emotional response, *alliesthesia-* pleasure	Frustration, resignation, dislike, indifference, anger, anxiety, regret, joy, happiness, etc.				
	Occupant´s Social Context	Common believes/expectations		High/low expectations			[5]	Focus group
		Social values/perceptions/preconceptions		Ethics, status, necessity, health, sustainability, environmental impact, etc.				Survey - Focus group - Observation logbook

After selecting the required parameters for the fieldwork, the following step is to determine the spaces to be measured and the population to be involved in the study. It is advisable to choose the greatest possible variety of classroom orientations (facing north, south, east or west) and vertical positions within the building (ground level, middle levels or top-level), aiming to ensure a good representation of the comfort conditions within the whole school. It is also important to have a balanced sample of students from diverse age groups and perform the surveys at different times of the day. The next step is to prepare the selected tools and formats and to liaise with the project stakeholders, which may include school directives, administrative and teaching staff, as well as parents. The aim here is to introduce and coordinate in advance all the activities to be carried out during the information-gathering period. It is advisable to conduct most activities with the support of the school´s teaching staff, as students are more familiar with them and tend to respond better to their directions. Additionally, giving students a brief and informal introduction on the basis and importance of thermal comfort helps to keep them engaged. For this purpose, it is crucial to previously undertake a training session with all the supporting team and prepare the teaching material and techniques according to the age of the children.

#### Planning

During the planning stage, it is advisable to create a relationship with the stakeholders of the school, so all the community is invested in the study. The administrative staff is usually very interested and open since the results of the TC studies could be of great use for the institution. In order to start developing the case study, it is usefull to gather all the available building drawings (plans and sections), school functioning information (class schedules, classroom uses, special activities, etc.), and school population (students per classroom and educational levels). This information allows the researcher to create a general picture of the school.

Following this stage, site visits are a crucial issue because they allow the researcher to confirm the information gathered from the building and the occupants. Taking pictures during the visits can help researchers make informed decisions at a later stage, for example, when choosing the best classrooms to measure.

Before starting the gathering phase, it is advised to have a previous meeting with all the school teachers and parents to explain and justify the study. The teachers play a very important role, since they are in charge of the classrooms, and they can raise awareness among the students about the importance of TC. When students are engaged, they tend to be more helpful, and there is less temptation to touch or tamper with the equipment. Additionally, informing parents directly about the study can make them more prone to participate and authorise their children to get involved too. If a parents meeting is not possible, then it is important to get all the mandatory authorisations using the school protocols. The lack of parental authorisations at this stage can generate problems or prevent gathering information from children.

### Gathering

**Fig. 2:**
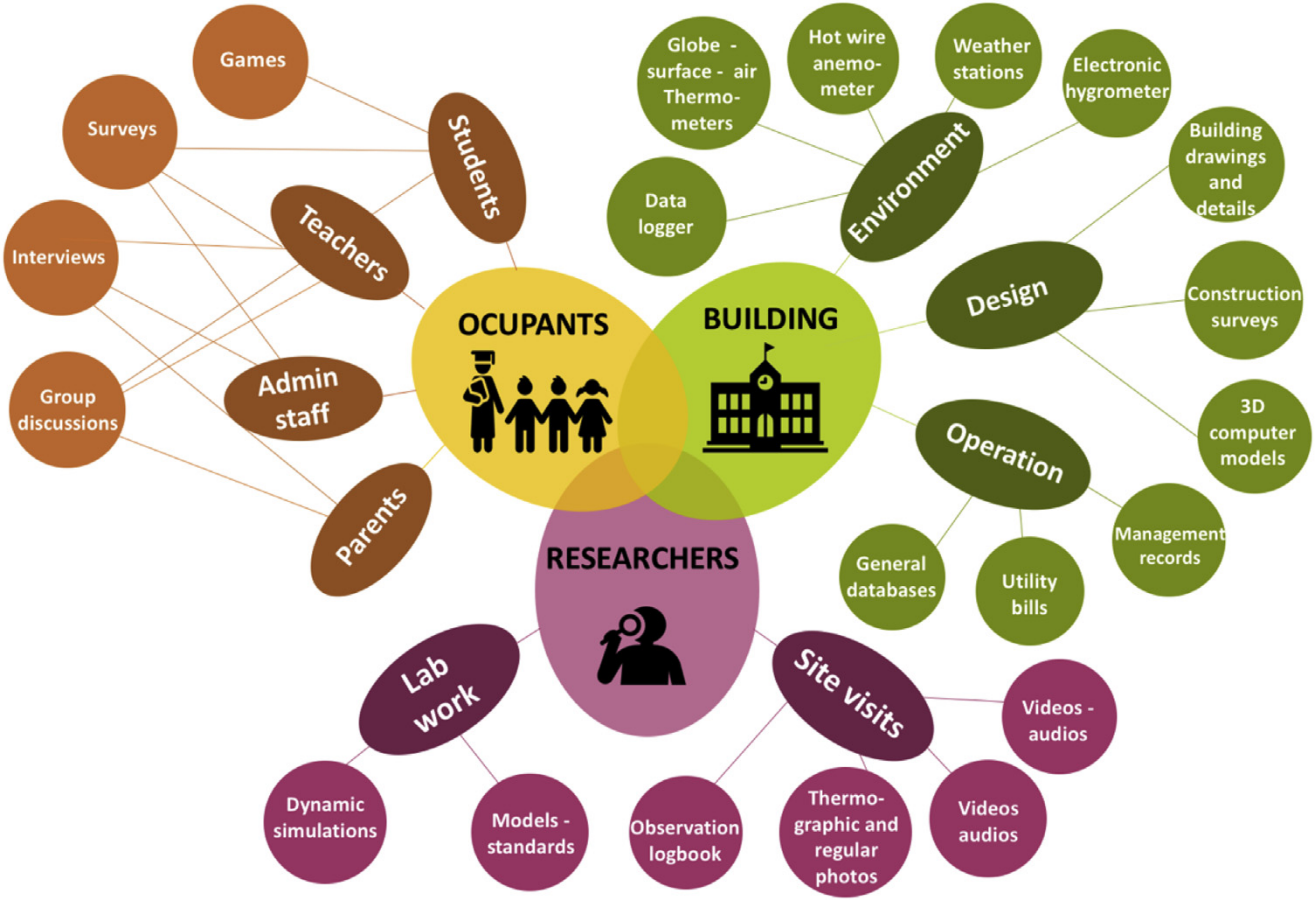
Data sources and collecting tools.

The *gathering* stage in this method involves four different steps: *Physical measurements*, *surveying*, *activities with focus groups*, and *observation and statistics*. These stages aim to collect information primarily from three sources: the building, the occupants and the researchers (see Fig. 2).

Most thermal comfort studies concentrate on measuring aspects of the building related to the physical environment. Classroom-comfort-data supports the collection of other elements associated with the design and operational characteristics of the building. It also proposes collecting information from different occupants via not only surveys but also interviews and activities with focus groups. The researchers or data-collectors play a very active role during fieldwork in the proposed method, by systematically inspecting on-site parameters related to the architectural design, as well as analytically observing the behaviours of the occupants.

#### Physical measurements

**Fig. 3:**
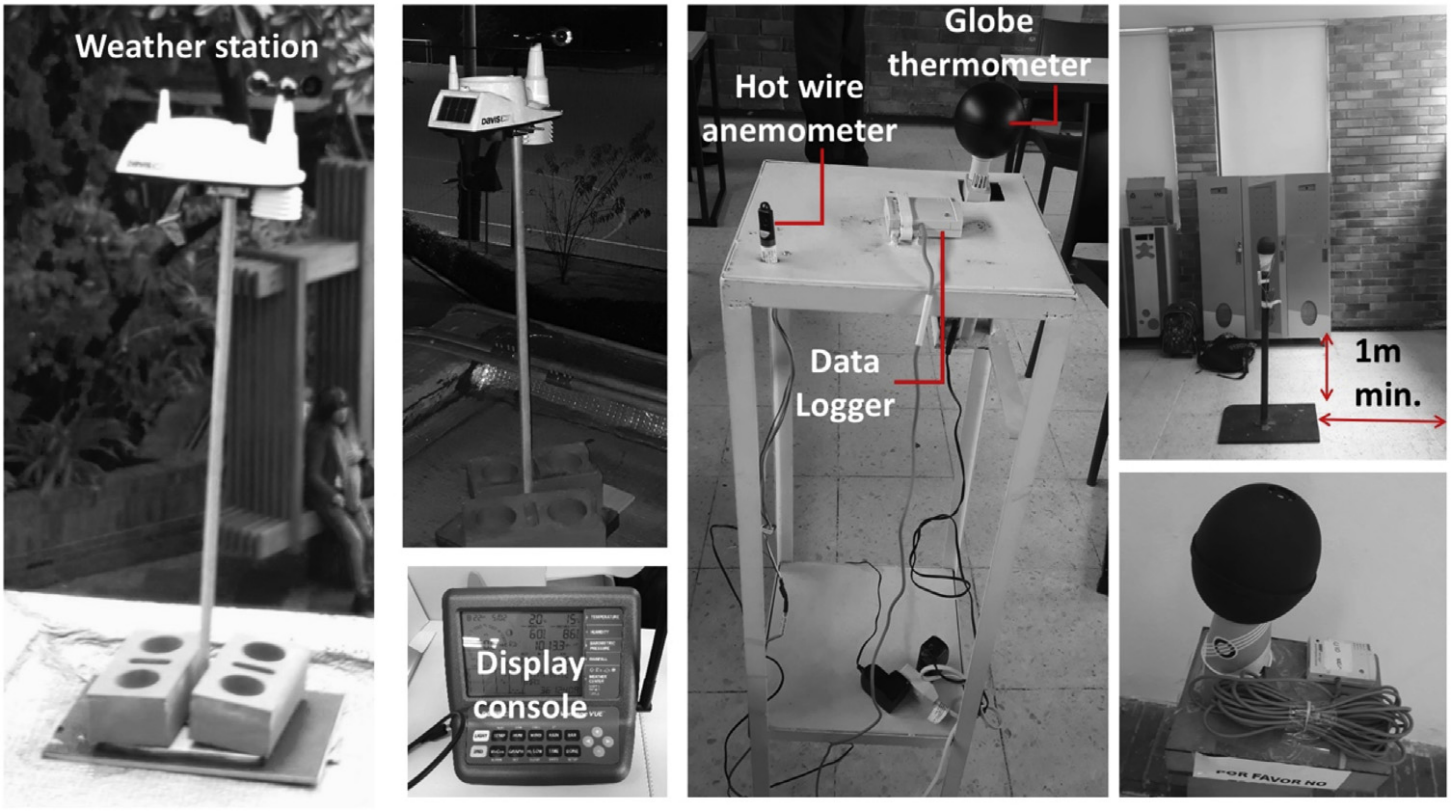
Examples of equipment to use for physical measurements.

Measuring the building’s physical conditions is often one of the most challenging tasks to achieve due to the economic limitations of acquiring all the necessary tools. There are various types of equipment to measure the physical space parameters listed in Table 1 (see Table 1). In the case of a school classroom, it is advisable to use the smallest size and amount of equipment possible to avoid interfering with teaching activities or to prevent accidents. Multi-channel data loggers are suitable options, as they record time-based information, are relatively small and can measure different parameters simultaneously (see Fig. 3). For example, some data loggers can record dry-bulb air temperature and relative humidity at the same time or use external probes to measure globe temperature, CO_2_ concentration, airspeed or air temperatures at different heights to determine stratification. Table 1 indicates the recommended equipment´s range and accuracy depending on the parameter.

It is advisable to use a portable weather station to record the prevailing mean outdoor temperature. The weather station can account for specific microclimatic characteristics of the location and provide more accurate results. It should be placed on a building´s rooftop, away from any obstacles. If there are no rooftops available inside the school, it is advisable to find a suitable location as close as possible to the measured spaces. If using this type of equipment is not an option, then data from the nearest available weather station should be acquired.

The ANSI/ASHRAE Standard 55 recommends taking measurements “in locations where the most extreme values of the thermal parameters are observed or estimated to occur (e.g., potentially occupied areas near windows, diffuser outlets, corners, and entries)” or “at a representative sample of locations where the occupants are known to, or are expected to, spend their time” … “If occupancy distribution cannot be observed or estimated, then the measurement locations shall include both of the following: a. the centre of the room or space b. 1.0 m (3.3 ft) inward from the centre of each of the room’s walls. In the case of exterior walls with windows, the measurement location shall be 1.0 m (3.3 ft) inward from the centre of the largest window” [9]. During the case studies, it was observed that children are often curious about the equipment and are tempted to touch it or interact with it. Therefore, locating it at the centre of the room was found to be inconvenient and unsafe, in some cases, especially for long-term measurements. Instead, it is advisable to place the equipment at the back of the classroom, especially if it is going to be left for a number of days. Ideally, place it 1.0 m away from the wall and far away from any infrared radiation (IR) or heat sources such as direct sunlight, incandescent lamps, radiant heaters, televisions, computers or projectors. The data from this equipment can be complemented with short-term measurements taken with additional equipment at the centre of the room during supervised visits, for example, at the time of the surveys.

Regarding the equipment height, the Standard 55 advises air temperature and average airspeed to be measured at the 0.1, 0.6, and 1.1 m above the floor for seated occupants and 0.1, 1.1, and 1.7 m for standing occupants. Additionally, it suggests operative temperature or the PMV model be calculated at the 0.6 m for seated occupants and 1.1 m for standing occupants. These recommendations are based on the proportions and body dimensions of adult occupants. No specific recommendations were found in the available literature for the case of children. Therefore, a short literature review on children anthropometric dimensions was carried out to establish the appropriate equipment height for the study of TC in classrooms with the Classroom-comfort-data method. The focus of this review was on Latin-American children; hence, the results may vary for children from other backgrounds.

Literature shows that, generally, the most sensitive human body-part to temperature is the face, as it has a high concentration of thermoreceptors for both cold and warmth [56,57]. Consequently, it makes sense to measure the conditions of the room at this height. Table 2 illustrates the average heights for 6-24-year-old occupants when standing and when seated, as well as eye-level when standing and nose level when seated. According to this information, Table 2 indicates the recommended equipment heights for each studied group when standing and when seated (see Table 2). It is advisable to complement this with measurements at other heights and locations in order to study stratification, asymmetries, and drafts within the space.

**Table 2:** Average anthropometric dimensions of Latin-American children, based on information from [58].

Group (years)	Weight (kg)			Total height standing (mm)			Eye-level when standing (mm)			Total height when seated(mm)			Nose level when seated (mm)			Recommended equipment height (mm)	
	Female	Male	Group	Female	Male	Group	Female	Male	Group	Female	Male	Group	Female	Male	Group	Standing	Seated
6	22.4	22.8	22.6	1167	1175	1171	1064	1067	1066	924	929	1853	802	805	804	1000	800
7	25.1	25.8	32.9	1218	1134	1322	1114	1120	1216	961	966	1043	836.5	840.5	913	1200	900
8	28.4	29.3		1269	1279		1166	1171		1000	1004		874	876.5			
9	32.3	32.8		1318	1334		1226	1226		1041	1045		912.5	913.5			
10	36.3	36.3		1399	1381		1185	1272		1091	1075		958	943			
11	42.3	40.6		1447	1437		1353	1327		1133	1113		996.5	978.5			
12	45.6	42.7	51.8	1500	1480	1560	1390	1369	1448	1163	1144	1211	1025.5	1010	1071	1400	1000
13	48.6	49.4		1533	1542		1421	1427		1195	1195		1056.5	1056.5			
14	53.1	55.5		1555	1611		1446	1494		1223	1256		1084.5	1112.5			
15	54.2	65		1577	1685		1472	1568		1222	1291		1078.5	1143.5			
16	56.4	65.6	61.6	1588	1700	1642	1479	1581	1530	1231	1309	1270	1084.5	1158.5	1122	1500	1100
17	57.4	66.9		1582	1705		1472	1587		1228	1309		1085	1161			
18	54.9	68.1		1572	1707		1468	1591		1224	1316		1079.5	1163.5			
19-24	55.5	68.2	61.9	1586	1709	1648	1478	1595	1537	1237	1320	1279	1091.5	1169.5	1131	1500	1100

According to the Standard 55, the lengths of the measurements “shall represent a sample of the total occupied hours in the period selected for evaluation (year, season, or typical day) or shall take place during periods directly determined to be the critical hours of anticipated occupancy” [9]. Measurement intervals of five minutes or less are suggested for dry-bulb air temperature and mean radiant temperature, and three minutes or less for humidity. Battery life and data storage capacity are important considerations when choosing equipment and when planning the frequency in which measurements need to be downloaded. It was observed during the case studies that data loggers measuring in intervals lower than 5 min might fill up before the measuring period is over.

#### Surveys

**Fig. 4:**
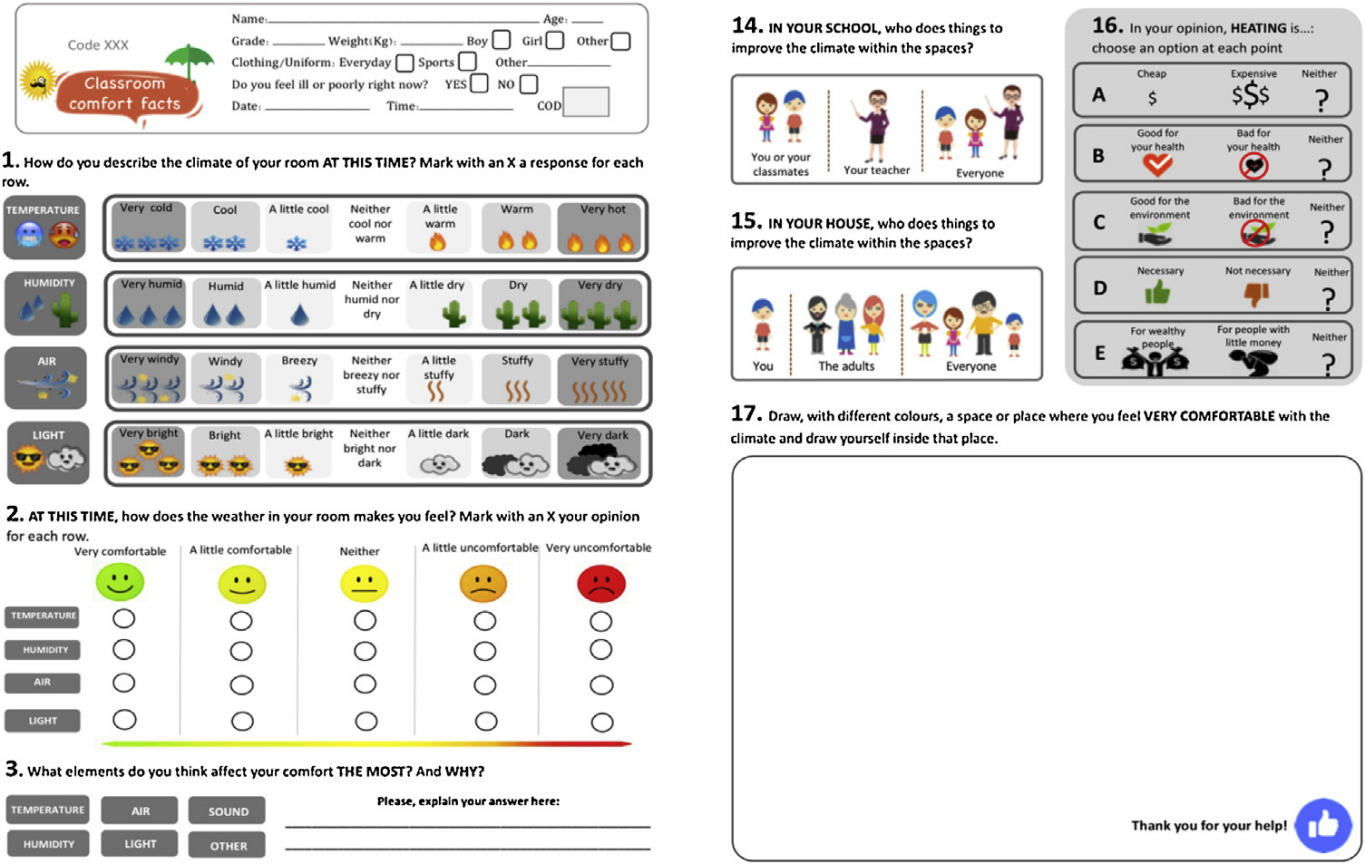
Examples of paper surveys.

This model suggests five types of surveys, which are included here as Supplementary material (see example in Fig. 4). These were designed according to the occupant’s age and development stage: Middle childhood (7–11 years old), early adolescence (12–15 years old), adolescences and young adults (16+ years old), teachers and staff, parents. These classifications were guided by the widely recognised Erik Erikson’s Theory of Psychosocial Development, which considers sociocultural determinants that influence human´s cognitive and emotional growth.

Depending on the targeted audience, the surveys can be implemented on paper or online. Paper surveys are convenient to use with young children or where there is no easy access to digital devices. Online surveys, developed with tools such as Google Forms or SurveyMonkey, are useful to reach parents or administrative staff. All surveys comprised an introduction with general questions and a body of 17 main questions aiming to collect a variety of data (see Table 3).

**Table 3:** Main questions in the surveys.

Questions		Type of Question and Choices	Data Aimed to Collect
GENERAL INFO	Name, age, course, weight, height, gender, clothing, general health	**Open-ended.**	Occupant physical characteristics.
	Date and time	**Open-ended.**	Fieldwork methodology.
**1.** How do you describe the climate of your room AT THIS TIME? Mark with an X a response for each row.		**Rating scale.** Rows: Temperature (very cold, cool, a little cool, neutral, a little warm, warm, hot.). Humidity (very humid, humid, a little humid, neutral, a little dry, dry, very dry). Air (very windy, windy, breeze, neutral, a little stuffy, stuffy, very stuffy). Light (very bright, bright, little bright, neutral, little dark, dark, very dark).	**Evaluation.** Thermal comfort sensation TSV during measurements (point-in-time). Based on the ASHRAE scale.
**2.** AT THIS TIME, how does the climate in your room make you feel? Mark with an X your opinion for each row.		**Rating scale.** Rows: Temperature, humidity, air, light. Columns: Very comfortable, a little comfortable, neither comfortable or uncomfortable, a little uncomfortable, very uncomfortable.	**Evaluation**. Thermal comfort vote TCV during measurements (point-in-time). Based on the Bedford scale.
**3.** What elements do you think affect your comfort the most?		**Closed-ended.** Temperature, humidity, air, light, other (which one).	**Perception.** Causality.
**4.** NORMALLY At what time of the day do you feel MOST COMFORTABLE in your classroom? Mark with an X your opinion for each row.		**Ranking order.** Rows: Temperature, humidity, air, light, other (which one). Columns: Early in the morning, midday, afternoon, all day.	**Perception.** General thermal comfort vote TCV/time.
**5.** If you feel UNCOMFORTABLE with the climate of your classroom, what happens? Mark your answer with an X.		**Multiple choice - single answer.** You enjoy the class more, carry on as normal, you have difficulty concentrating, other (which one).	**Perception.** Conduciveness – helps or hinders the goal.
**6.** What do you like the MOST and the LEAST about the climate of your classroom? Or What would be your ideal thermal environment? (the second option is more suitable for older children or adults)		**Open-ended.**	**Preference**. Value and expectation.
**7.** The climate of YOUR CLASSROOM makes you feel:		**Multiple choice.** Happy, comfortable, proud, surprised, indifferent, sad, angry.	**Perception.** Emotional response.
**8.** When you are UNCOMFORTABLE with the climate of your classroom, what DO YOU DO TO FEEL BETTER? Mark with an X the option or options that you prefer.		**Multiple choice - multiple answers. 1:** Open the window, close the window, open the curtain, close the curtain. **2:** Take off some clothes, put on some clothes, drink a cold beverage, drink a hot beverage, use a hand wave fan, splash yourself with water, move to get warm, stay still. **3:** Get used to it. **4:** Exit the classroom.	**Adaptive strategies** **1.** Environmental modifications to space. **2.** Behavioural adaptations. **3.** Expectation adjustment. **4.** Withdraw from space.
**9.** Circle the place that you think is the MOST COMFORTABLE in the climate of your city:		**Multiple choice - single answer.***Drawings of the same place varying the following elements:* a water fountain, vegetation, curtains, windows, mechanical fan, rug, heating, chimney.	**Perception.** Common believes and preconceptions.
**10.** If you could CHANGE something in YOUR CLASSROOM to feel more comfortable, WHAT WOULD YOU CHANGE?		**Multiple choice - multiple answers.** The material of the walls, the material of the roofs, the windows, the lighting, the odour, the noise, the location of the room, the height of the room, the volume of the room.	**Adaptive strategies** Environmental modifications to space – preconceptions.
**11.** If you could ADD something in YOUR CLASSROOM to feel more comfortable, WHAT WOULD YOU ADD?		**Multiple choice - multiple answers.** Heating, air conditioning, mechanical fans, curtains or blinds, plants.	**Adaptive strategies** Environmental modifications to space- preconceptions.
**12.** Compared to your classroom, YOUR HOUSE is:		**Multiple choice.** Much colder, a little colder, the same, a little hotter, much hotter.	**Physical-cognitive context** Points of reference – expectations.
**13.** The climate of YOUR HOUSE makes you feel:		**Multiple choice.** Happy, comfortable, proud, surprised, indifferent, sad, angry	**Physical-cognitive context** Points of reference – emotional response.
**14.** IN YOUR ROOM, who does things to improve the climate within the spaces?		**Multiple choice.** You or your classmates, your teacher, everyone, nobody.	**Social context** Perceived control- agency.
**15.** IN YOUR HOUSE, who does things to improve the climate within the spaces?		**Multiple choice.** You, the adults, everyone, nobody.	**Social context** Points of reference – Perceived control- agency.
**16.** In your opinion, HEATING is: choose an option at each point		**Likert-type scale. 1**.cheap, expensive, neither. **2.**Good for your health, bad for your health, neither. **3.** Good for the environment, bad for the environment, neither. **4.** Necessary, not necessary, neither. **5.** For wealthy people, for people with little money, neither.	**Social context**. values/perceptions/preconceptions.
**17.** Draw, with different colours, a space or place where you feel VERY COMFORTABLE with the climate and draw yourself inside that place.		**Open-ended.**	**Physical-cognitive context** Points of reference – expectations.

The question´s language, graphics, and complexity were adjusted in each survey according to the ages of the targeted groups. During the case studies, it was noticed that for some young children, the meaning of terms such as *slightly, neutral, unacceptable or satisfied* is not always clear. Therefore, the terminology was changed to words such as *little*, *neither X or Y, uncomfortable* or *happy*. Illustrations were included for most questions to aid comprehension. The complexity of the question can be increased according to the student´s age. For example, for questions 7, 10, 11, 13, and 15 occupants over 12 years old can be asked to explain the reasons behind their answer briefly. In question 17, older occupants were given the option of describing the space where they felt very comfortable, instead of drawing it. The parent´s survey included only nine questions (1, 2, 3, 5, 8, 10, 11, 13 and 16) considered relevant for this group. The wording of these questions was changed to evaluate the parent´s perception and expectations over the comfort of their children. The surveys included here were applied in classrooms located in a mild tropical climate, for other types of climates and locations, the answer-choices for questions 8–11 and 16 can be adjusted if needed to suit other common practices.

Surveys graphics should be designed in the clearest way possible. For example, they should only include one or two questions per row and avoid cramming too many questions on the same page. This allows small children to read and answer all the questions properly. Otherwise, there is a risk that children may get visually tired and skip some of the questions. Graphics and spaces should be big enough for small children to read, write and draw properly.

The ANSI/ASHRAE Standard 55 advises that “surveys shall be solicited from the entire occupancy or a representative sample thereof. If more than 45 occupants are solicited, the response rate must exceed 35 per cent. If solicited occupants’ number between 20 and 45, at least 15 must respond [33–75 per cent]. For under 20 solicited occupants, 80 per cent must respond” [9]. Usually, the number of students in a classroom can vary from 5 to 50 depending on the school´s teaching methodologies and economic recourses. However, ethical guidelines in many countries state that minors can only answer surveys with the signed authorisation from their parents or guardians. Therefore, to follow the Standard 55´s recommendations, fieldwork studies in schools should aim to survey a larger percentage of occupants than studies in other types of buildings.

**Fig. 5:**
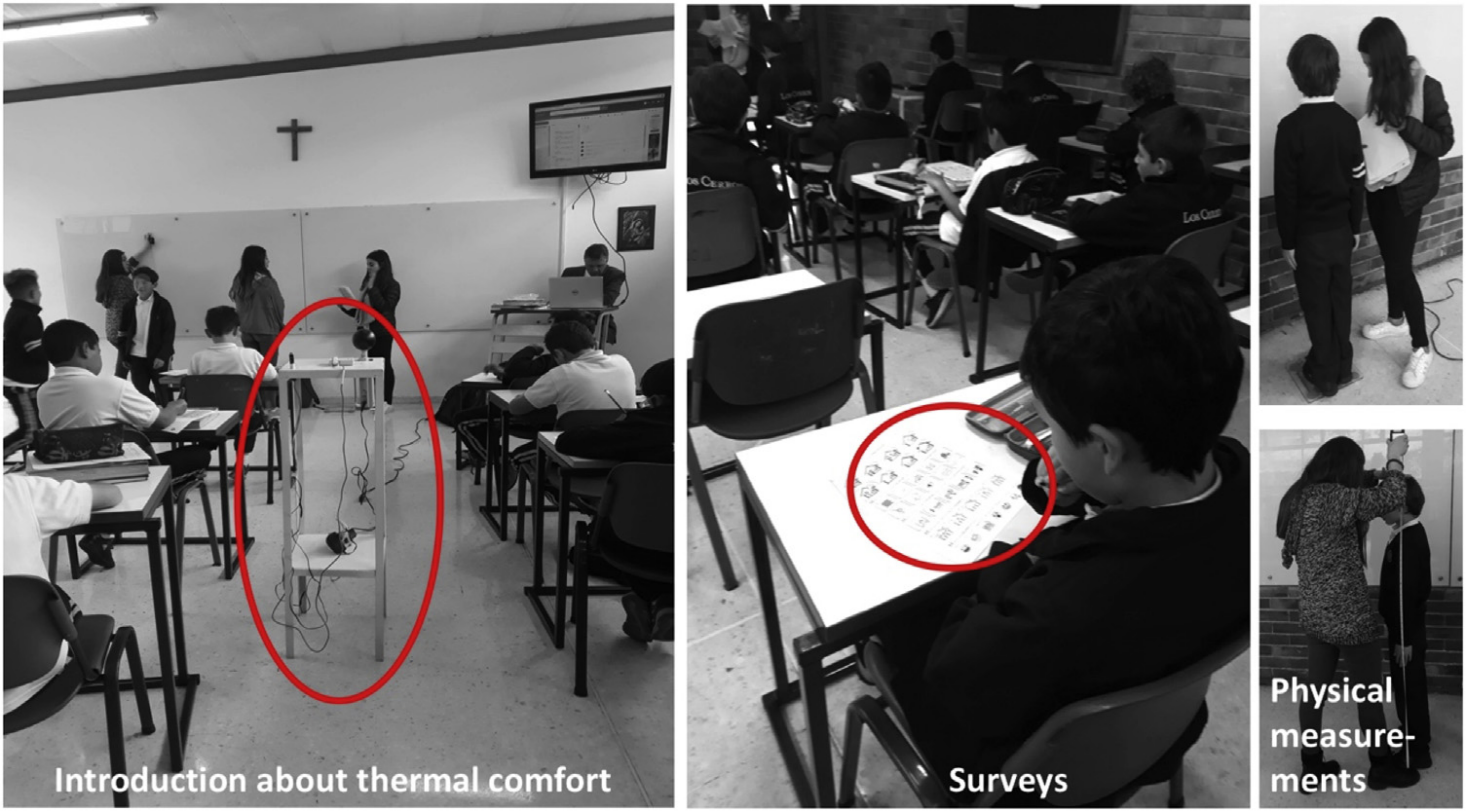
Surveys and other parallel activities.

While students are answering the survey, other activities can be performed, such as taking student’s physical measurements, running other activities with focus groups or filling out the observation logbook (see Fig. 5).

#### Focus groups

Focus groups and one-to-one interviews are widely used strategies to gather qualitative data on human experiences. These offer a more interactive setting than a survey and encourage a more engaging discussion with other participants. It is argued here that, in the context of thermal comfort studies, focus groups and interviews are powerful tools for the understanding of social preconceptions, expectations, opinions, points of reference and added values. These tools can help to uncover correlations between various thermal comfort parameters and identify differences between age groups. For example, younger children may be prevented to open the windows or may have to ask their teachers before opening them, but older children may have the agency to do it without asking. Additionally, other parameters not always taken into account in the surveys, such as outside noise, may affect the adaptive actions taken by the students in order to improve their indoor comfort.

The Classroom-comfort-data method suggests collecting information from at least one focus group (5–10 participants) in each student´s age category. Activities with the focus groups can vary according to age, from games with younger children to group discussions with older participants. One-to-one interviews are generally more suitable to use with administrative staff or parents. Table 4 illustrates examples of questions that can be employed to structure the discussion during the focus group activities (see Table 4). These were designed to complement the questions of the surveys, placing emphasis on aspects related to occupants’ behaviour and social context. Focus groups with small children should last no more than 15 min since children tend to get tired or distracted quickly.

**Table 4:** Examples of questions to structure the focus groups.

Data Aimed to Collect	Question	Recommendations for the Researcher
Preconceptions Causality	• In your own words, what influences the climate in your classroom?	Adjust language according to the student´s age group. Influences: affect/impact/causes. Climate: weather/environment/ambience.
Conduciveness /goal	• Do you think that the climate in your classroom affects your health? • Do you think that the climate in your classroom affects your wellbeing/feelings? • Do you think that the climate in your classroom affects your studies?	Ask participants to explain their answers and if necessary, give examples to encourage discussion. Avoid being bias by including positive and negative examples of answers such as the climate makes you happy/sad, energetic/tired, enthusiastic/bored, or you experience any change.
Evaluation and perception	• Which words can you use to describe the climate of the classroom? • Do you think that the climate in your classroom is comfortable, uncomfortable or in between?	Ask participants to explain their answers.
Common believes	• What positive aspects and what negative aspects can be said about the climate of your classroom?	Try to list both positives and negatives without being bias.
Adaptive strategies	• If change is needed, what do you think could help to improve the climate of a classroom?	If necessary, give examples to encourage discussion (changing the windows, adding vegetation, using a fan, etc.) Try to include examples of both passive and active strategies.
Perceived agency	• Who should implement/do these changes?	Examples: you, the adults, your teacher, the school, etc.
Perceived control	• If you feel uncomfortable with the climate of your classroom, what could you do?	Examples: put more clothes on or take clothes off, take cold or hot drinks, stay still or do nothing, etc.
Expectation	• How could you describe the ideal climate for a classroom? • How would the ideal climate make you feel?	Examples: no particular feeling, proud/embarrassed, pleased/unhappy, etc.
Perceived value, status	• What do you need to have the ideal climate for a classroom?	Examples: nothing because you already have it or you would need creativity, money, time, help, etc.

#### Observations and statistics

**Fig. 6:**
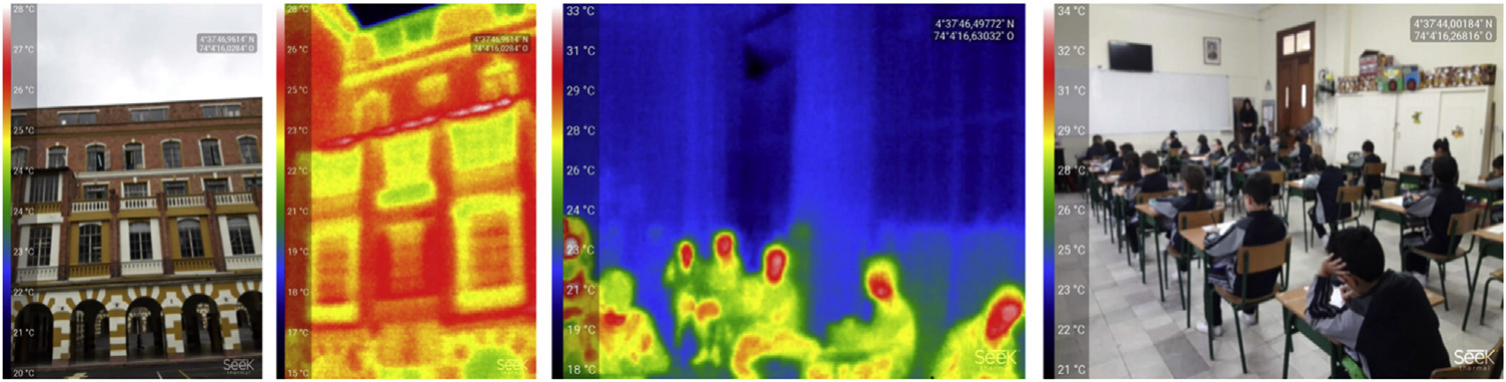
Regular and thermographic photographs showing temperature variations on the building façade and the occupant´s bodies.

Fieldwork often includes observations from the researchers or people collecting data. This method highlights the significance of this information by specifying strategies to acquire it and organise it. These strategies comprise the use of visual media such as thermographic photographs, regular photographs, audio recordings and drawings (see Fig. 6).

**Fig. 7:**
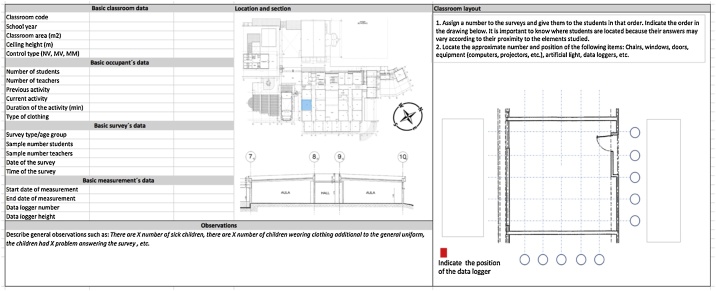
Examples of an observation logbook FM-01-CRR.

Structured observation-logbooks are also of great use (see example in Fig. 7). They help to simplify the length and design of the surveys because many ordinal questions (e.g. room orientation, time of the year, room location within the building, the positioning of the children, etc.) can be answered by the observer instead of the participant. This is very relevant in the case of school fieldwork as children’s attention span is commonly shorter compared to adults, and they tend to get more tired or bored with long questionnaires. Additionally, observing on-site activities through the critical and trained eye of the researcher may uncover unusual or incidental issues that can influence the study of thermal comfort or have an impact on data collection. For example, students may have difficulty understanding a question, some participants may feel unwell during the activities or the data logger might be moved. Researchers are the ideal individuals to observe and record aspects related to the architectural design of the studied space, such as the envelope materials and building components, as well as the location of potential thermal bridges. However, in some cases, it is necessary also to have independent data collectors that are skilled in the subject to avoid bias.

Additional statistical information from school records or other available databases can be gathered to determine operational aspects of the building related to energy consumption, equipment usage or occupancy schedules. Researchers can also produce valuable information to complement the fieldwork, for example, by constructing dynamic simulations of the building, which serve to generate information that is difficult to obtain from other sources. Dynamic simulations and charts can be developed based on the building drawings, with software such as EnergyPlus™ coupled with a graphical interface such as DesignBuilder. These tools allow the assessment of characteristics such as the facade insulating properties or equipment heat gains. Furthermore, dynamic simulations produced in advance are very useful to complement or validate the estimated thermal performance of the building against the real conditions found on-site.

### Presentation

#### Selection and pre-processing

**Fig. 8:**
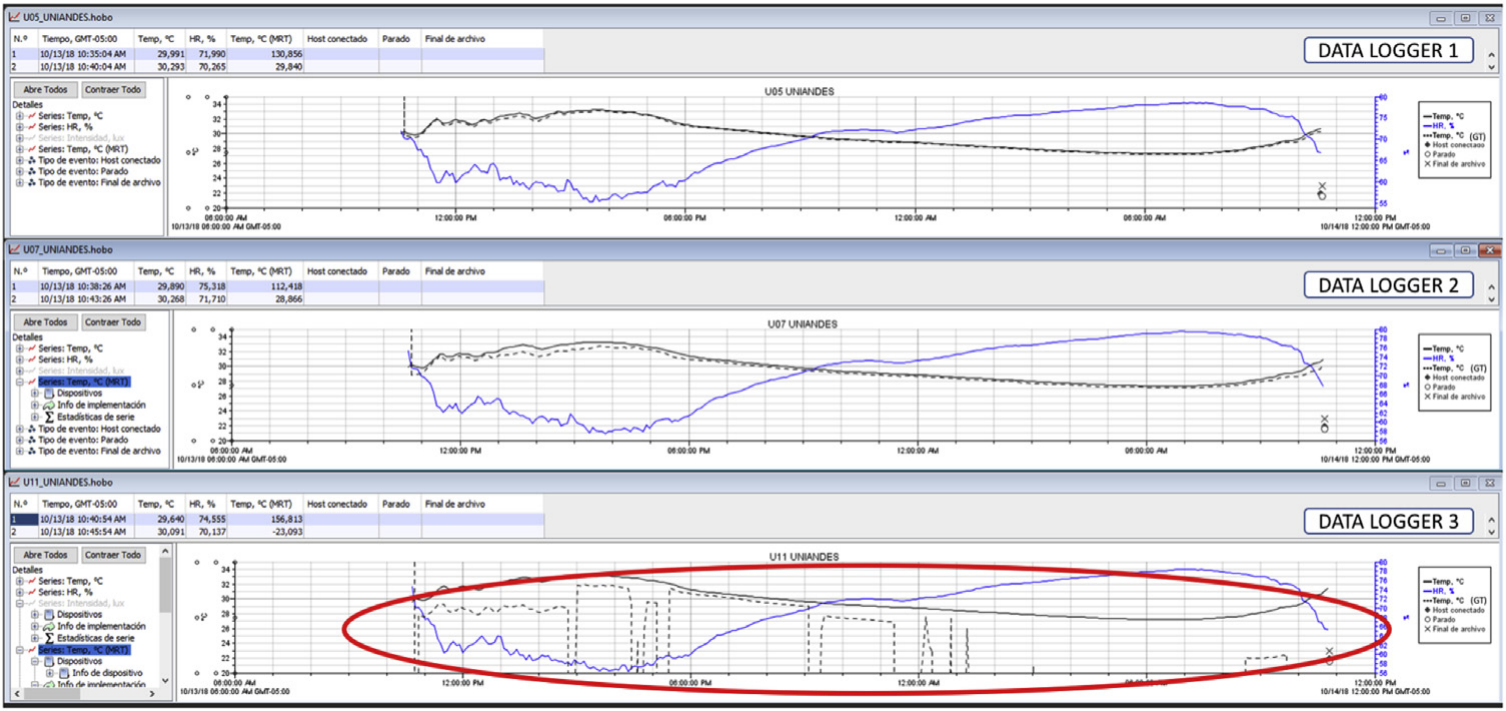
Example of common errors in the environmental measurements.

The final stage of the Classroom-comfort-data method involves filtering and refining the collected information. This can be done with the use of Excel spreadsheets and pivot tables or with another data-mining software. Creating these tables can be time-consuming; however, they allow the researcher to quickly identify potential inaccuracies or errors within the data and broadly visualise emerging trends and patterns. Running quick graphs with the data logger´s software can also help to detect unusual outcomes or missing information. Fig. 8 shows an example of simultaneous recordings with three data loggers, which reveals errors in the collection of GT from data logger 3 (see Fig. 8).

The purpose of this step is not to fully analyse the data, but to prepare it for further study with one or more assessment models. It is advisable to pre-process data as soon as possible after the gathering stage, in case any measurement needs to be retaken while the equipment is still on-site.

## Method validation

As mentioned before, Classroom-comfort-data was tested, refined and validated through two different case studies in schools in Bogota, Colombia. These were carried out at different times of the year (March-June and August–November). Bogota is located within the tropics (4.7 °N, 74.1 °W), at a regular altitude of 2600 m above sea level, which contributes to creating cold climatic conditions (Cfb climate type in the Köppen-Geiger classification). There are little seasonal variations throughout the year in terms of average temperature (±14 °C) and relative humidity (±73 %), and there are two marked rainy periods (April-May and October-November). The selected schools differed from each other in terms of building features, classroom occupancy, pedagogical approach, and occupant’s characteristics. The first case study was a four-storey school building erected during the early 1940s and comprised of 37 classrooms, laboratories, offices and special classrooms for art and music classes. The rooms were 62.5 m^2^ on average with 3.7 m height ceilings and placed around two courtyards in a traditional cloister style. This school had a mix-gender population mainly from low and middle-income families and with an average occupancy of 32 students per classroom. The students stayed in the same space during most of their classes with a fairly traditional teaching format. The second case study was a single-storey building constructed in the 1960s with 13 classrooms. The rooms were 72 m^2^ on average with 3 m height ceilings and placed in a U-shaped block along internal corridors, leaving one paved courtyard in the middle. This was a boys-only school with a population mainly from middle and high-income families and with an average occupancy of 25 students per classroom. The students had activities in different classrooms with more active teaching and learning format. Students wore uniforms in both of the studied schools; therefore the clo values were established from observations in the logbooks. Adjustments would need to be made to the surveys for schools where students wear other clothing. Both case studies were buildings of cultural interest for the city due to their architectural value. According to local architectural magazines from the 1960s, the second school was designed taking into account climatic variables.

**Fig. 9:**
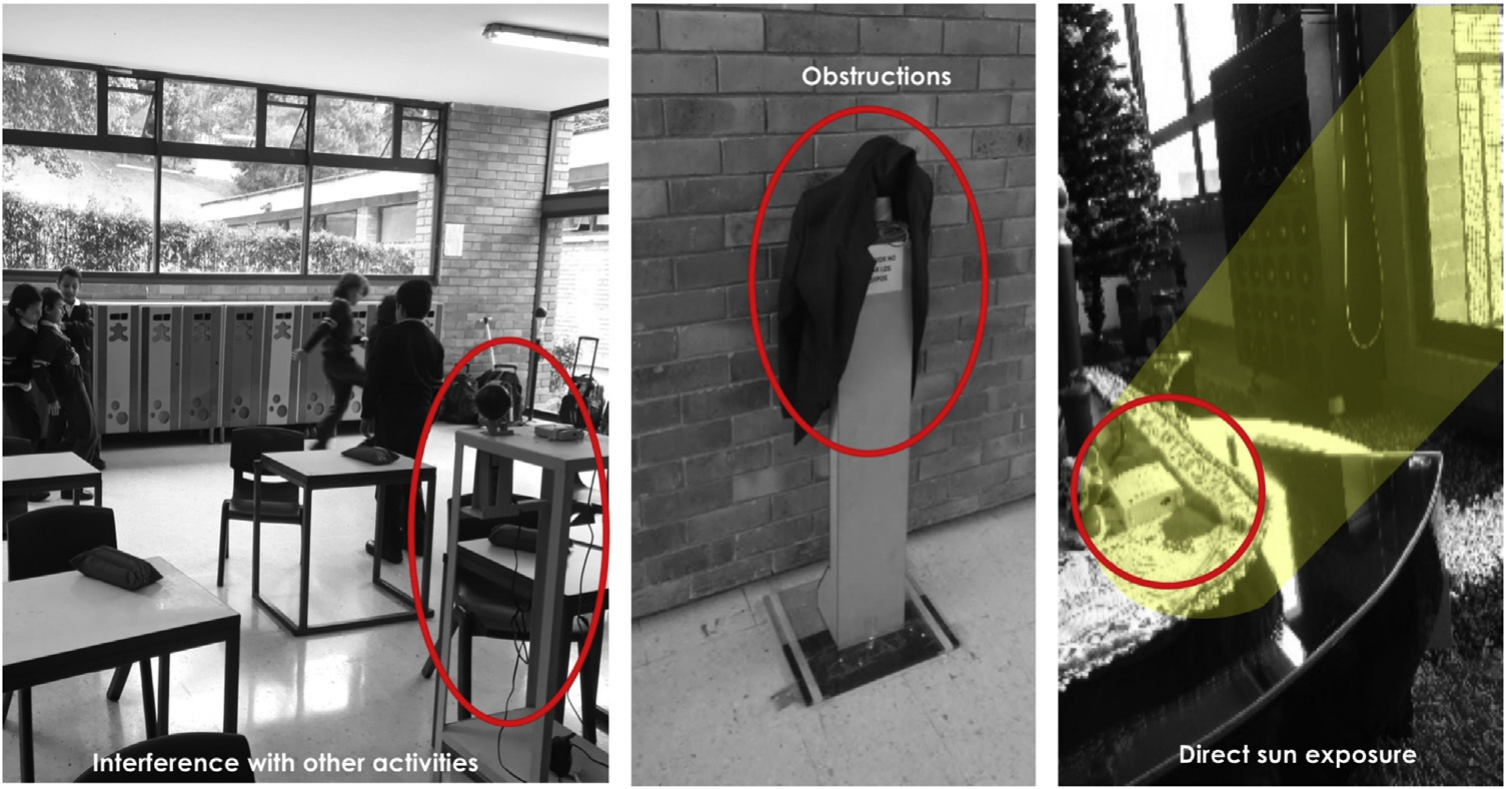
Examples of potential problems or limitations encountered during fieldwork in schools.

With the first experience, valuable lessons and feedback were acquired regarding the method´s general structure, procedures, and resources. The techniques and formats were adjusted on-the-go to suit unforeseen events during the classes, confirming that unlike fieldwork in other types of buildings, school classrooms present additional challenges related to the nature of children´s behaviour (see Fig. 9). This opportunity served to fine-tune the method for further testing and improvement during the second case study. In further research by the authors, the data gathered during the case studies was subsequently analysed with both, the static and adaptive methods, as well as with a theory of environmental satisfaction and human comfort developed by Shin [41] and a model based on cognitive appraisals by Keeling and others [43]. These types of assessments provided worthy information that led to significant conclusions in the case of the classrooms used as case studies. For example, it was noticed that, for young children, acceptable temperature levels were not always indicators of thermal comfort. Other aspects such as the room layout and the location of windows have more impact on their perceive sensation of comfort.

## Conclusions

The method Classroom-comfort-data presented here has shown to be a suitable and flexible method for gathering comprehensive TC data in school classrooms within different contexts. It has proven to complement the data-collecting techniques proposed in the existing standards regarding scope, range, adaptability and means.

Classroom-comfort-data facilitates the collection of a wider range of physiological, psychological, and social parameters during fieldwork (up to 49 parameters). For example, useful information regarding the occupants´ evaluation, perception and preferences for comfort, the adaptive strategies used by them to achieve comfort and the physical-cognitive and social context that influences their choices. This extends the scope of a typical post-occupancy thermal comfort study based on the current standards, which normally focuses on 2–5 parameters. With the information gathered via this method, a more comprehensive and holistic analysis can be achieved by combining the existing standards with alternative models to assess environmental satisfaction [41,43].

The tools and procedures suggested with the Classroom-comfort-data method, offer diverse means and ways to collect information, which can be adapted according to particular characteristics of climate, building design, occupancy levels, and cultural contexts. The method can be adjusted and adapted to different contexts and conditions within educational buildings, making it suitable to be replicated by professionals and researchers in the field of TC. The measuring tools indicated in the standards (e.g. thermometers, anemometers, and data loggers) are coupled with other widely available and affordable tools such as portable weather stations and thermographic camares for mobile phones. The method suggests complementing the physical and environmental information collected with all these tools with dynamic simulations to create a more accurate picture of the existing conditions. Additionally to surveys, other structured procedures are proposed in this method, such as observation-logbooks, audio-visual aids, interviews and activities with focus groups.

The use of the Classroom-comfort-data method has the potential to support or encourage future lines of research regarding the shift from universal standards towards more tailored standards. This shift is already on sight with the emergence of alternative regional assessment models for specific tropical regions in Southeast Asia [59], México [60] and Brazil [2], and other models for residential buildings in different climatic zones of eastern China [61] or office buildings in hot and humid climates of India [62]. However, the accuracy of these theoretical models relies significantly on the quality of the recorded data from real buildings. Deficiencies in data collection and fieldwork methodologies are one of the biggest challenges faced in thermal comfort research, as well as limitations in geographic coverage. The method proposed here aims to contribute towards improvements in these areas, with special attention to educational environments.

Gathering information on influential but currently overlooked parameters that affect thermal comfort could give new insights into the subject and improve the existing algorithms suggested by the standards. For example, relative humidity is not a central parameter for the adaptive model, but it is a defining feature in the tropics, being particularly high in humid tropical climates and extremely low in tropical desert regions. Another important parameter is the altitude, which defines distinct climates in mountain ranges, as atmospheric pressure at high altitude varies significantly during the day [13]. Altitude also affects the oxygen concentration in the body and the function of the vascular system, resulting in changes in metabolic rates. Therefore, the impacts of altitude on the perception of thermal comfort are likely.

The effects of air quality and air movement on thermal comfort are also in need of further research. For example, CO_2_ levels are often overseen as a core parameter in thermal comfort. However, they can be found in relatively large concentrations within AC spaces and densely populated urban environments, both common scenarios in tropical regions. High levels of CO_2_ have been associated with an over-stimulation of the respiratory system, resulting in increased metabolic rates and heat exchange with the environment, which suggests potential effects on thermal comfort. Another area of interest is the degree of the agency that occupants have to adapt their environment or chose personalised means of heating, cooling or ventilation. Agency is thought to influence psychological adaptation significantly [63].

The present work centres on thermal comfort in classroom environments, therefore it has limitations regarding the study of related subjects (e.g. acoustic comfort, visual comfort and air quality), which can also impact physical and mental health, learning processes and productivity in the occupants of these buildings. Therefore, it may be combined or improved with other collecting methods for parameters in these areas, according to the focus of the post-occupancy study.

Declaration of Competing Interest

The authors declare that they have no known competing financial interests or personal relationships that could have appeared to influence the work reported in this paper.
