# Supplementary material for: Classroom-comfort-data: A method to collect comprehensive information on thermal comfort in school classrooms
Source: MethodsX. 2019 Nov 7;6:2698–719. doi: 10.1016/j.mex.2019.11.004 (PMC6881643; doi:10.1016/j.mex.2019.11.004)
Supplement: Supplementary file 1 [file mmc1.docx]

**Supplementary Material**

| **Item** | **Description** |
| --- | --- |
| SR01 / 7-11 | Occupants´ survey for the middle childhood groups (7-11 years old) |
| SR02 / 12-15 | Occupants´ survey for the early adolescence’s groups (12-15 years old) |
| SR03 / 16+ | Occupants´ survey for the adolescences and young adults’ group (16+ years old) |
| SR04 / T | Occupants´ survey for teachers and teaching staff |
| SR05 / P | Occupants´ survey for parents |
